# Injuries and illnesses related to dinghy-sailing on hydrofoiling boats

**DOI:** 10.1186/s13102-021-00343-8

**Published:** 2021-09-30

**Authors:** Francesco Feletti, Eric Brymer, Matteo Bonato, Andrea Aliverti

**Affiliations:** 1 Department of Diagnostic Imaging, Ausl della Romagna, S. Maria delle Croci Hospital, Ravenna, Italy; 2 Faculty of Health, Southern Cross University, Gold Coast Campus, Lismore, Australia; 3 Department of Biomedical Sciences for Health, Università degli Studi di Milano, Via Giuseppe Colombo 71, 20133 Milan, Italy; 4 IRCCS Istituto Ortopedico Galeazzi, Milan, Italy; 5 Dipartimento di Elettronica, Informazione e Bioingegneria, Politecnico di Milano, Milan, Italy

**Keywords:** Tendinosis, Sports trauma, Epidemiology, Injury prevention, Physical exercise, Dinghy-sailing

## Abstract

**Background:**

Hydrofoil technology has changed sailing, significantly increasing its speed and resulting in spectacularity and mass media interest. Although high speed can expose participants to a risk of high-energy trauma, there are no scientific studies related to trauma in dinghies that exploit this technology. Therefore, this study aims primarily to measure the injury rate, and identify the kind and anatomical distribution of most common injuries and secondarily identify the traumatic dynamics most often involved and the main risk factors.

**Methods:**

This descriptive epidemiology study examined data relative to injuries and illnesses suffered by 77 sailors (91% males) from 13 nationalities during three international, gathered through a specifically designed questionnaire.

**Results:**

The prevalence of illnesses and overuse injuries during the regatta week were 6.5% and 18.2%, respectively, while the incidence of acute injuries was 16/1000 sailor-hours. Upper limbs, lower limbs and lumbar spine were involved in 34.6%, 26.9% and 15.4% of cases of musculoskeletal injuries, respectively. None of the acute injuries reached the maximal score of severity, while the higher score value was 63/100. Most of the reported illnesses (80%; n = 4) were upper respiratory tract infections with a prevalence in the week of the regatta of 5.2% and an incidence of 0.51/1000 sailor-hours. Environmental factors played a role in 77% of the incidents resulting in acute injuries.

**Conclusions:**

This study provides valuable information for regatta organizers, boat builders, athletes, coaches, and doctors. In addition, it fosters the importance of ergonomics of boats, adequate clothing and specific physical training for injuries and illnesses prevention.

**Supplementary Information:**

The online version contains supplementary material available at 10.1186/s13102-021-00343-8.

## Background

Competitive dinghy sailing is practiced at varying levels, from amateur competitions to the Olympics. Dinghies range in size and complexity from single-handed 2.4 m Optimist, intended for use by children, to 49er, the Olympic two-handed skiff-type high-performance sailing dinghy with a total sail area of 59.2 m^2^ and capable of speeds easily exceeding 20 knots. According to the recent review by Nathason, sailors at risk of acute injuries, overuse injuries, environmental injuries, and sailing-related illnesses [1]. Injury rates at an international 2014 Olympic-class regatta and among elite dinghy racers in New Zealand have been reported as 0.59 per 1000 h and 0.2 injuries per year, respectively; while in dinghies mixed population, injuries were 4.6 per 1000 days of practice [2–4]. In dinghies, most acute injuries are contusions (9–55%) commonly caused by falls and collision with the boat during mismanaged maneuvers [1]. Head trauma is among the most severe reported injuries and is often caused by the boom’s impact. Hand injuries are also common (6–31%) and mainly include lacerations, cuts and fractures from handling lines or the center-board. Capsizing and collisions with other boats or objects are other frequent mechanisms of injury. Overuse injuries especially are mainly represented by low back pain (29–45%) and knee pain (13–22%) and are common especially among Olympic-class sailors. High winds, turning maneuvers (i.e. tacks, jibes), operator inattention and inexperience have been identified as leading contributing factors for injuries [1].

Over the last decade, hydrofoil technology has had an enormous impact on sailing, providing high speed and spectacle that attract media attention and increase sailing popularity. While foil sailing was introduced with the Nacra 17 class in the 2016 Rio de Janeiro Summer Olympic Games, hydrofoil technology is also used in various amateur sailing classes. In particular, the 36th edition of America’s Cup, which took place from 6th to 21st of March 2021, in Auckland, New Zealand, included the AC75 catamarans [5]. Despite the advantages offered by hydrofoils, the technology exposes crews to high speeds and accelerations (over 40 knots, 46 mph, 74 km/h) that increases the risk of high-energy trauma, especially in the case of collisions between boats or boat capsizing [6]. In hydrofoil technology, the shape of the appendices’ lamina diverts the water flow downwards, thus exerting an upward force on the lamina. This upward force lifts the boat’s keel, decreasing water drag and allowing for an increase in speed. The speed, in turn, increases the apparent wind, contributing to the possibility of exceeding the actual wind speed and achieve significantly higher final velocities than traditional boats. High speed can expose participants to a risk of high-energy trauma, as some events reported by the media seem to confirm. For example, in 2013, Artemis Racing’s AC72 jumped (somersaulted) and broke apart while training for the 34th America’s Cup. The accident resulted in a crewmember’s death who suffered blunt trauma, severe head injuries and cuts and drowned after being trapped underwater [7]. In 2017, during a practice race for the Nacra 17 World Championships in France, the US crew pitch-poled and capsized at a speed of 17 knots resulting in the helmsman partially losing three fingers of the right hand [8]. However, the media often highlight outdoor sports risks after a severe accident or fatality [9]. However, it is possible that most injuries are minor and that while the speed and maneuverability increase the risk of injuries, they are not necessarily serious.

Sailing on foiling boats requires fine coordination between the crewmembers to maintain precarious flight. Furthermore, wind shifts and water movements also affect the flying boat’s speed and balance, continually challenging the flight. A recent study by Terrien et al. [10] showed that the natural environmental condition influences the interaction between the crewmembers and the Nacra-17 class boat. Moreover, extra-personal and interpersonal coordination processes are also distinct cognitive and interactive process influences to maintain or restore flight stability, and reflective interpersonal coordination processes to anticipate environmental events or understand past events [10]. Since hydrofoil has only recently been introduced into the sport of sailing, to date, no scientific studies are investigating the risk of acute injuries, overuse injuries, environmental injuries, and sailing-related illnesses associated with this technology. Therefore, this study aimed to provide preliminary information about the most common injuries related to hydrofoil sailing. The primary outcome was to classify the lesions and identify which anatomical parts may be affected. The secondary outcome was to determine the traumatic dynamics most often involved and the main risk factors to guide specific recommendations.

## Methods

### Study design

This descriptive epidemiology study was conducted following the Strengthening the Reporting of Observational Studies in Epidemiology (STROBE) [11] during three different regattas: 2018 and 2019 Foiling Week editions held in Malcesine (Italy) and the Moth World Championship held in Perth (Australia) in December 2019. The recruitment of participants was extensive in Foiling Week 2018. We preliminarily sent an informative e-mail to all pre-registered sailors; furthermore, upon completion of regatta registration, participation in the study was proposed in person by one of the researchers; finally, during the regatta briefing, the organizers explained the aims of the research and encouraged participation. As for the other two regattas, the participants were allowed to participate, but the information campaign was in charge of the organizing committee only and took place in a sparse and non-systematic way. Before entering the study, the participants were fully informed about its aims and procedures. Furthermore, each participant released written informed consent before the inclusion in the study. The study protocol was approved by the Ethical Committee of Politecnico di Milano (Prot. 2/2018), with current National and International laws and regulations governing the use of human subjects (Declaration of Helsinki II).

### Data collection

The study protocol required completing the same questionnaire pre and post the sailing event to distinguish between injuries sustained during the regatta from those before the regatta. The questionnaire was written in English and designed specifically for the study (supplementary material 1). As well as demographic and sailing class information, the two-part questionnaire adopted a symptom-focused approach, validated by Clarsen et al. [12] for gathering data on injuries and illnesses. This approach has proven effective for capturing data about injuries in other outdoor sports, such as kitesurfing [13, 14]. Initially, participants were asked to respond to the four following questions: “Have you had difficulty sailing due to health problems (injuries/illnesses)?” (Question 1); “To what extent have you reduced your sailing volume due to health problems over the past week?” (Question 2); “To what extent have health problems affected your performance over the past week?” (Question 3); “To what extent have you suffered from sports-related symptoms in the past week?” (Question 4). An affirmative answer to one of the four questions triggered further questions aiming to identify sailing-related injuries and classify them regarding the anatomical site and type of lesion according to ICD-10 [15]. For this purpose, we asked participants to respond to a predetermined list of possibilities addressing both quantitative and qualitative elements. We encouraged a complete and detailed description of the type and severity of the injury. Another part of the questionnaire concerned the event’s dynamics: participants were asked to give a detailed account of the incident’s dynamics and causes. The causes of the incidents were analyzed using the Haddon matrix, a model broad in scope that proved to be adaptable to extreme sports contexts [16, 17]. Participants involved in more than one incident were asked to respond to the questionnaire for each event. A physician was available in person or by phone to help participants complete the survey. In addition, the physician conducted in-depth anamnesis to ensure that the data collected was detailed, accurate and classified appropriately. Inclusion criteria for the sailors were the participation in one of the regattas mentioned above, having provided written informed consent to participate in the study and answering at least the first part of the second questionnaire (questions 1, 2, 3, 4). Exclusion criteria were the participation in foiling watersports on hydrofoil vehicles other than sailing boats. In the data analysis, we excluded medical conditions that had a declared different origin than the practice of hydrofoil sailing. Questionnaires were available in both paper and electronic form, published on the website of the association Extremesportmed (https://extremesportmed.org/survey/performance-test-for-the-foiling-week/) using the software Gravity Form 2.3.22.6.

### Data analysis

The data relative to the second administration of the questionnaire were extracted and analyzed, specifically: sex, date of submission and all the data required to localize and characterize each reported clinical condition anatomically. The responses to the four initial questions were scored according to Clarsen et al. [18] to calculate a severity score from 0 to 100 for each reported problem. Specifically, questions 1 and 4 were scored 0-8-17-25, and questions 2 and 3 were scored 0-6-13-19-25. For each participant reporting a problem, the pre-regatta questionnaire was also checked to define whether the disorder was already present. Still, the calculation of the incidence excluded these data due to the lack of an appropriate denominator. We obtained data about nationality and boat class by matching the questionnaire data with those provided by the regatta organizers. The regatta organizers then offered data relating to the number of heats run and the average duration of the races. Data was transferred from the website onto Excel datasheets and analyzed using Wizard Pro 1.3.27 and JASP 0.14.1 software. The epidemiological analysis was based on the International Olympic Committee consensus statement [19]. Incidence was calculated based on the injuries reported in the second questionnaire not registered in the first one, which reasonably occurred during the regatta. Active sailing time was determined by assessing the average duration of each race based on the number of the disputed heats and considering a mean time for each heat of 20 min (data provided by the organizers). This data was subsequently used as the denominator. Data analysis examined possible relationships between the occurrence and type of injuries and the type of boat using the X2 test. Descriptive statistics were used to explore the data relative to the dynamics of the sustained injuries. Confidence intervals related to injury rates were assessed using the Wilson score interval method.

## Results

Limitedly to the Foiling Week 2018 regatta, a participation rate of 44.8% was calculated. In total, 78 sailors (91% males) from 13 nationalities took part in the study; 60 participants were from the 2018 Foiling Week, three from the 2019 foiling week regatta and 15 from the Moth World Championship. According to the exclusion criteria, one of the participants was excluded because of practicing foiling kitesurfing, and our analysis included the data relative to 77 participants. Two participants sent the second questionnaire multiple times without any difference in the contents, and we excluded repetitions, including the last invoice only. One injury was excluded as it was sustained from a different sport (skiing). A majority of the participants, 75.5% (n = 58), sailed single-handed boats (namely the Moth class, A class, Waszp and S9cat). In comparison, 19.5% (n = 15) sailed crew dinghies, both double-handed (Nacra 17, Flying Phantom, and prototypes) and 2.5% (n = 2) on three-person boats (Etf26); the data relative to the boat was missing in two cases. The days from the last regatta’s day and questionnaire completion were missing data in seven of the cases collected in 2019. Based on the available data, the mean interval was 2.21 days.

Overall, 26 (33.7% of the participants) reported 28 medical conditions, 14 overuse injuries, nine acute injuries and five illnesses, with two sailors reporting two distinct medical conditions each.

Eleven of the reported conditions were already present in the week before the regatta. When considering all medical conditions, the prevalence in the regatta week was 36.4%, with an incidence of 36/1,000 sailor-hours. The prevalence of illnesses and overuse injuries during the regatta week were 6.5% and 18.2%, respectively, while the incidence of acute injuries was 16/1.000 sailor-hours.

**Fig. 1:**
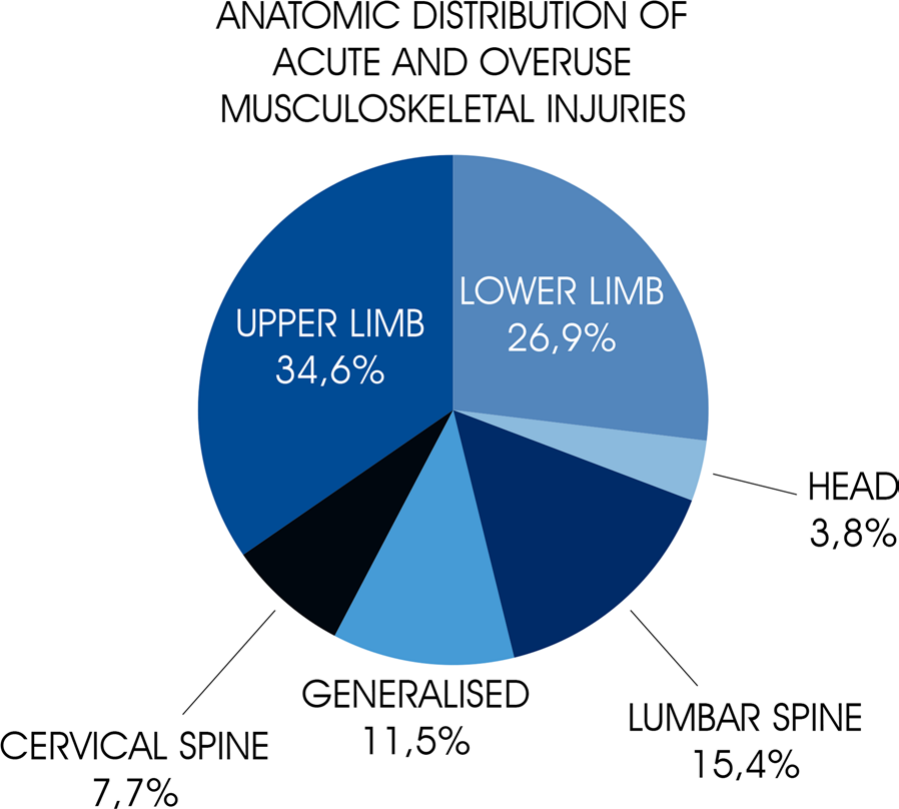
Anatomic distribution of acute and overuse musculoskeletal injuries during the regatta week

The breakdown of injuries sustained in each of the studied competitions is reported in Table 1. The 17 conditions during the studied regattas, included seven acute injuries, seven overuse injuries, and three illnesses. Musculoskeletal injuries were mainly distributed to the upper limbs (34.6%; n = 9), followed by lower limbs (26.9; n = 7) and lumbar spine (15.4; n = 4) (See Fig. 1).

**Table 1:** Number of injuries and hours of active sailing in each of the studied competitions

Regatta	Participants	Reported conditions	Actual days of regatta	Number of disputed heats	Hours of active sailing	incidence (medical conditions/h)	IC (95%)
Foiling week 2018	61	12	6	18	244	0.04918	0.0284–0.0840
Moth World Championship 2019	15	5	4	12	60	0.08333	0.0361–0.1807
Foiling week 2019	3	0	4	12	18	0.00	0.0000– 0.1759

A mean duration for each strike of 20’ was estimated by the organisers of the regattas

Most of the reported illnesses (80%) were upper respiratory tract infections with an incidence of 0.51/1000 sailor-hours and a prevalence in the week of the regatta of 5.2%.

**Fig. 2:**
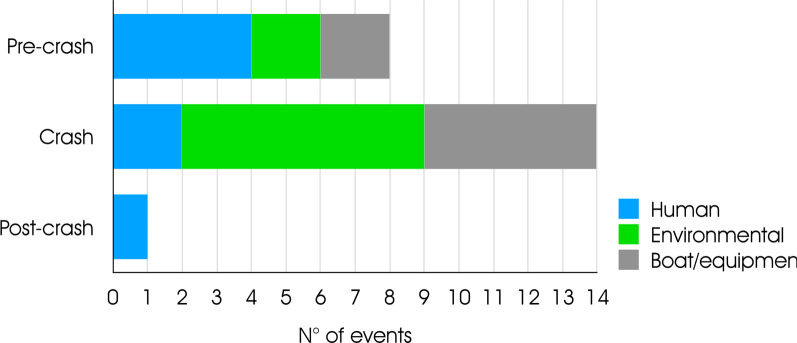
Acute injuries and their dynamics anc causes during the regatta week classified according to the Haddon Matrix

Acute injuries and their dynamics and causes classified according to the Haddon matrix are reported in Table 2. Environmental-related factors during the crash phase were the leading contributing factors (See Fig. 2).

**Table 2:** Acute injuries: classification and dynamics

Injury	Anatomic region	Severity Score	Dynamics	Haddon matrix		
				Human	Environmental	Boat and equipment
Strain	Right Axilla	63	Nose diving, collision with the shroud	*Pre-crash* Limited experience with foiling boats experience Lack of training for agility *Crash* Violation of rules (obstruction)	*Crash* Strong wind	*Crash* Collision with the shroud
Contusion/ bruising	Thigh	28	Impact on wing during maneouver	*Pre-crash* Limited experience with foiling boats experience *Crash* Lack of attention *Post-crash* Lack of knowledge of what to really do in case of a crash	*Pre-crash* Field of regatta conditions *Crash* Strong wind	*Crash* Collision with the terrace
Contusion /bruising	Hand	8	Contact with cleats during jibing	*Pre-crash* Lack of training for agility	*Crash* Strong wind	*Crash* High speed of the boat and of the manouvre (jibe)
Cut /Laceration	Thigh	8	Being thrown forward while jibing. Impact with forward Strutt/ Shroud plate	–	*Crash* Strong wind (15 Knots)	*Pre-crash* Hard boat surfaces. Solid wing bars and vang strut. *Crash*: Equipment failure
Contusion/bruising	Head Shoulder	8	Broken wand caused a nose dive resulting collision with the mast base and compression bars	*Pre-crash* Lack of physical training (fatigue)	–	*Pre-crash* Lack of ergonomic forward strutt or shroud plate
Contusion/ bruising	Upper arm	8	Contact injury with the Boom	–	*Crash* Strong wind	–
Fracture	Forearm and Wrist	22	Contact with the boat	–	*Crash* High waves	–
Whiplash	Cervical spine	28	Boat nose diving, Projection outside the boat hard landing on water	–	*Pre-crash* Field of regatta conditions (waves from motor boats) *Crash* Strong wind	–
Ligament rupture	Ankle	20	Contact with the boat (immobile parts)	–	–	*Crash* Equipment failure

Overuse injuries and illnesses and their causes are reported in Table 3.

**Table 3:** Overuse injuries and illnesses: classification and cause or contributing factor/s

Condition	Anatomic region	Severity Score	Cause or contributing factor/s related to foiling-boat sailing
*Illnesses*			
Infection	Upper respiratory tract	8	Probably caused by rapid variation in temperature (windchill)
Ophthalmological and otological inflammation	Eye and ear	8	Unspecified
Infection	Upper respiratory tract	8	Unspecified
Infection	Upper respiratory tract	20	Unspecified
Infection	Upper respiratory tract	8	Probably caused by rapid variation in temperature (windchill)
*Overuse injuries*			
Lumbosciatalgia	Lumbar spine/Lower back/Lower extremity	27	Recurrence of symptoms from previous injury (Lumbar disc herniation during sailing in Laser class sailing).
Lumbosciatalgia	Lumbar spine/Lower back/Lower extremity	14	Inadequate athletic preparation (fatigue)
Cervicalgia	Cervical spine	12	Recurrence of symptoms from previous injury
Pain, ache and soreness (DOMS)	Musculoskeletal system	8	Inadequate athletic preparation(fatigue)
Muskuloskeletal pain ache and soreness (DOMS)	Musculoskeletal system	6	Unspecified
Nerve injury/spinal cord injury	Lumbar spine/lower back, Upper extremity	22	Unspecified
Tendinosis/tendinopathy	Shoulder	27	Strong wind
Tendinosis/tendinopathy	Knee	20	Strong wind
Cuts Laceration/abrasion/skin lesion	Hand Finger	6	Wet environment, high tension on the mainsheet, mainsheet too thin
Exhaustion and dehidration	Musculoskeletal	28	Inadequate athletic preparation (fatigue)
Muscle cramps or spasm in the evening after many hours of practice	Forearm/hand	23	Inadequate athletic preparation (fatigue)
Nerve injury	Lumbar spine	6	Field of play conditions. Inadequate athletic preparation (fatigue)
Blisters	Hand	6	Inadequate technical preparation
Pain/Lateral overload	Knee	8	Inadequate technical preparation (Wrog jibing technique)

## Discussion

This study aimed to classify the most common injuries related to hydrofoil navigation, which anatomical parts could be affected and the most common traumatic dynamics to guide specific recommendations.

Analyzing the demographics of our series, we found a disproportionately higher percentage of males; however, this data is expected in sailing; apart from the Olympics, where the female participation rate is 50%, among the most significant events in the sailing calendar, the percentages span between zero and 24%. Traumatic injuries were calculated at a rate of 0.035 (SE: 0.024) per hour of active practice [20]. An injury rate of between 0.29 and 5.7 per 1.000 h was reported for dinghy sailing in a relatively recent review. However, that study also included data from the Nacra 17 class in its first configuration. Despite not being a genuinely foiling boat, it was equipped with curved fins that may be considered forerunners of foiling technology, capable of providing vertical lift under certain sailing conditions. In a review of sailing accidents more broadly, Nathanson [4] reported an injury rate of 4.60/1.000 practice hours. In our series, the injury rate was significantly high and seemed to confirm that foiling increases injury risk. All but one of the reported acute injuries resulted from the collision with fixed and mobile parts of the boat: shroud, wing bars, vang strut, cleats, and the boom. One case of whiplash was caused by deceleration caused by the boat nose-diving in the presence of winds generated by motorboat traffic in the regatta field. These dynamics suggest that speed and acceleration play a vital role in injury generation on foiling boats.

However, in our study, none of the acute injuries reached the maximal score of severity as determined by the four grading questions (max. score recorded: 25), while the higher reached score value was 63/100. This indicates that while foiling technology might increase the frequency of injuries, the injuries sustained are not necessarily severe injuries. Most reported acute injuries are unspecific trauma generated by collision with the boat and the equipment; collision with boat parts is a common cause of injuries also in traditional sailing. However, the increased acceleration forces that may result in a higher rate of foiling boats’ injuries suggest some specific preventive measures. While in traditional dinghy sailing, strong wind is reported as a cause of injuries in novices, Nathanson et al. [4] found that strong wind was a cause in two-thirds of accidents resulting in injuries. Findings from this study suggest some interventions. The results linking high winds to accident and injuries suggest that careful consideration of the practice of foiling in extreme wind conditions is essential. Moreover, waves may increase the risk of a crash, and therefore the use of motorboats’ near the race site should be limited. Sailors should prepare themselves for hydrofoil sailing’s specific technical and dexterity requirements to cope with the increased boat speed and maneuvers’ high velocity. Equally, a high level of concentration is required to effectively steer these boats that require appropriate physical and psychological training to reduce fatigue. Specific medical and ergonomic guidelines should be prepared to encourage the adoption of rounded forms rather than sharp edges, especially for the deck and drift, while cleats, winches, and bailers should be positioned in places with reduced likelihood of accidental contact. Since drowning has been reported as the cause of 73.1% of recreational boating deaths among recreational sailors, life jackets should be worn at all times while sailing on any types of boats. The life jacket should provide head support to protect against drowning in case of head injury followed by a fall in the water [21].

Some overuse injuries were reported in this study, including hand lacerations, cervicalgia and sciatica, and tendinopathy. All these conditions have already been reported as common overuse conditions in traditional sailing [1, 4, 6, 22–26] However, one participant reported a specific traumatic mechanism resulting from an incorrect jibing technique, with the leg placed on the trampoline in a flat position with the bent knee resulting in knee pain due to lateral overload on the joint. Ensuring effective technique, optimizing posture, and strength training [24] could prevent knee injuries. Specific training should also be undertaken depending on the particular foiling class. Equally, a chilled body is more susceptible to injury of joints, tendons and muscles. Waterproof garments are especially useful in reducing heat dispersion, particularly in inclement weather and proved to be effective at preventing muscle tears and bursitis in the joints of the lower limbs [26]. The adoption of hardwearing, performance sailing gloves may prevent hand injuries. Their use is crucial in cold climate conditions and when handling ropes under high tension. Shrouds and ropes should be of the highest caliber and should be changed regularly to maintain their smooth and soft texture to avoid abrasions and lacerations to the hands. In our series, most reported illnesses were upper respiratory tract infections (URTI), with a weekly prevalence of 5.2%. URTI are common among sailors and have already been registered during America’s Cup, where illnesses accounted for 35% of all health problems suffered, the majority consisting of upper respiratory tract infections (40%) [24, 27]. Specifically, the data reported by Neville et al. [24] (a total of 129 wks of infection in 38 sailors, over 50wks) means a weekly prevalence during America’s Cup of 6.7% attributed to the immune system weakening resulting from physical exercise, psychological stress and cold and damp conditions. An adequate, balanced diet that maintains a positive energy balance and an adequate intake of carbohydrates, proteins and micronutrients may help prevent these infections [25, 27]. Stress levels should also be monitored by assessing markers of tiredness and overtraining [28]. Neville et al. [29] also proposed regular monitoring of resting s-IgA together with a simple fatigue questionnaire to determine the risk of upper respiratory tract infections in sailors.

This study has some limitations. The relatively small sample size and consequent law statistical power did not allow firm conclusions on the identification of typical injuries, anatomical distribution and main risk factors during hydrofoil sailing. Data collection during major international competitions was essential to collect relevant, consistent data under controlled circumstances. At the same time, however, it may have influenced decisions to participate in the study as crews were focused on the competition. The data collection method we used has already been shown to collect more sports-related medical conditions than traditional methods. Comparing the quantitative results of this study with the previous studies’ data must consider the possibility of differences related to the different method of data collection. This was a relatively short-duration study. Although longitudinal studies are needed, this study enabled collecting information about hydrofoil sailing that could be functional for further, more extensive studies. Finally, not all the participants were native English speakers, and this may have introduced bias due to a wrong interpretation of the questionnaire. However, we speculate that this may have limited effect on the present study because all sailors participated in international regattas, with sailing instructions and regulations in English. Good knowledge of the language is therefore assumed.

In conclusion, most of the reported musculoskeletal injuries were to the upper limbs (34.6%), among which one third were represented by hand contusions and lacerations, followed by shoulder strains and contusions.

This study provides potentially helpful information for regatta organizers, boat builders, athletes, and their coaches and doctors. The results also provide the basis for new studies. In hydrofoil sailing, much more than in traditional sailing, there should be a focus above all on the ergonomics of boats and specific physical exercises adapted for the crew training and rehabilitation of crews.

## Supplementary Information

**Additional file 1**. Questionnarie used in the study.

## Data Availability

The datasets used and/or analyzed during the current study are available from the corresponding author on reasonable request.
