# Supplementary material for: Injuries and illnesses related to dinghy-sailing on hydrofoiling boats
Source: BMC Sports Sci Med Rehabil. 2021 Sep 30;13:118. doi: 10.1186/s13102-021-00343-8 (PMC8485530; doi:10.1186/s13102-021-00343-8)
Supplement: Supplementary file 1 — Additional file 1. Questionnarie used in the study. [file 13102_2021_343_MOESM1_ESM.docx]

**Username:**

|  |
| --- |

**e-mail:**

|  |
| --- |

**Sex**

1. M
2. F

Required (single answer)

**Question 1**

Have you had any difficulties participating in normal training and competition due to injury, illness or other health problems LAST WEEK?

1. Full participation without health problems
2. Full participation, but with injury/illness
3. Reduced participation due to injury/illness
4. Cannot participate due to injury/illness

Required (single answer)

**Question 2**

To what extent have you reduced your training volume due to injury, illness or other health problems today?

1. No reduction
2. To a minor extent
3. To a moderate extent
4. To a major extent
5. Cannot participate at all

Required (single answer)

**Question 3**

To what extent has injury, illness or other health problems affected your performance today?

1. No effect
2. To a minor extent
3. To a moderate extent
4. To a major extent
5. Cannot participate at all

Required (single answer)

**Question 4**

1. To what extent have you experienced symptoms/health complaints today?
2. No symptoms/health complaints
3. To a mild extent
4. To a moderate extent
5. To a severe extent

Required (single answer)

**Did you report this problem previously?**

Yes/No

**Was this problem already being treated?**

Yes/No

**By whom? :** …

|  |
| --- |

**Please define whether the problems you referred was an illness or an injury**

1. Illness
2. Injury

Required (single answer)

*Enabled conditional logic (rules to dynamically display or hide this field based on values from another field.): hide this field if all of the following match:*

*Question 1 is “Full participation without health problems”*

*Question 2 is “No reduction”*

*Question 3 is “No effect”*

*Question 4 is “No symptoms/health complaints”*

**Illnesses-affected system**

1. Upper respiratory tract (nose, sinuses, pharynx, larynx)
2. Lower respiratory tract (trachea, bronchi, lungs)
3. Gastrointestinal
4. Cardiovascular
5. Urogenital, gynaecological or reproductive
6. Endocrine or metabolic
7. Haematological or immunological
8. Neurological, central nervous system
9. Dermatological/skin
10. Musculoskeletal
11. Dental
12. Ophthalmological/ontological
13. Psychiatric/psychological
14. Other

Required (multiple possible answers)

*Enabled conditional logic (rules to dynamically display or hide this field based on values from another field.): show this field if all of the following match:*

*Definition is “Illness”*

**Illness symptoms**

1. Pain, ache or soreness
2. Fever, excess sweating or chills
3. Nausea, vomiting or diarrhoea
4. Weight loss or dehydration
5. Fatigue, lack of energy, lethargy or arterial hypotension
6. Irregular heartbeat, palpitation, syncope, collapse or chest pain
7. Congestion, hypersecretion rhinorrhoea or discharge
8. Cough, wheezing, dyspnoea or shortness of breath
9. Dizziness or vertigo
10. Rash, itch or eczema
11. Numbness, weakness or tingling
12. Mood/sleep disturbance, anxious or depressed
13. Other

Required (multiple possible answers)

*Enabled conditional logic (rules to dynamically display or hide this field based on values from another field.): show this field if all of the following match:*

*Definition is “Illness”*

**Onset of illness**

1. Sudden onset
2. Gradual onset
3. Required (single answer)
4. Cause of illness
5. Pre-existing disease (exacerbations of allergy, asthma, diabetes, degenerative, etc)
6. Infectious (viral, bacterial, fungal, etc)
7. Environmental (heat, cold, altitude, etc)
8. Nutritional, endocrine or metabolic disturbance
9. Drug related or toxic reaction
10. Exercise related (dehydration, exhaustion, etc)
11. Psychiatric
12. Other/idiopathic

Required (multiple possible answers)

*Enabled conditional logic (rules to dynamically display or hide this field based on values from another field.): show this field if all of the following match:*

*Definition is “Illness”*

**Injury body part**

1. Face (including eye, ear, nose)
2. Head
3. Neck/cervical spine
4. Thoracic spine/upper back
5. Sternum/ribs
6. Lumbar spine/lower back
7. Abdomen
8. Pelvis/sacrum/buttock

Optional (multiple possible answers)

*Enabled conditional logic (rules to dynamically display or hide this field based on values from another field.): show this field if all of the following match:*

*Definition is “Injury”*

Body extremity

1. Upper extremity
2. Lower extremity

Optional (single answer)

*Enabled conditional logic (rules to dynamically display or hide this field based on values from another field.): show this field if all of the following match:*

*Definition is “Injury”*

**Upper extremity**

1. Shoulder/clavicle
2. Upper arm
3. Elbow (anterior/posterior)
4. Elbow (medial/lateral)
5. Forearm
6. Wrist
7. Hand
8. Finger
9. Thumb

Optional (multiple possible answers)

*Enabled conditional logic (rules to dynamically display or hide this field based on values from another field.): show this field if all of the following match:*

*Definition is “Injury”*

*Body extremity is “Upper extremity”*

**Lower extremity**

1. Hip
2. Groin
3. Thigh
4. Knee (anterior/posterior)
5. Knee (medial/lateral)
6. Lower leg
7. Achilles tendon
8. Ankle
9. Foot/toe

Optional (multiple possible answers)

*Enabled conditional logic (rules to dynamically display or hide this field based on values from another field.): show this field if all of the following match:*

*Definition is “Injury”*

*Body extremity is “Lower extremity”*

**Type of Injury**

1. Concussion (regardless of loss of consciousness)
2. Fracture (traumatic)
3. Stress fracture (overuse)
4. Other bone injuries
5. Dislocation, subluxation
6. Tendon rupture
7. Ligamentous rupture
8. Sprain (injury of joint and/or ligaments)
9. Lesion of meniscus or cartilage
10. Strain/muscle rupture/tear
11. Contusion/haematoma/bruise
12. Tendinosis/tendinopathy
13. Arthritis/synovitis/bursitis
14. Fasciitis/aponeurosis injury
15. Impingement
16. Laceration/abrasion/skin lesion
17. Dental injury/broken tooth
18. Nerve injury/spinal cord injury
19. Muscle cramps or spasm
20. Growth plate disturbance/avulsion
21. Other

Required (single answer)

*Enabled conditional logic (rules to dynamically display or hide this field based on values from another field.): show this field if all of the following match:*

*Definition is “Injury”*

**Mode of onset injury**

1. Sudden onset incident
2. Gradual onset incident

Required (single answer)

*Enabled conditional logic (rules to dynamically display or hide this field based on values from another field.): show this field if all of the following match:*

*Definition is “Injury”*

**Main cause of the injury**

1. Traumatic injury
2. Overuse injury

Required (single answer)

*Enabled conditional logic (rules to dynamically display or hide this field based on values from another field.): show this field if all of the following match:*

*Definition is “Injury”*

**Traumatic injury**

1. Contact injury
2. Non-contact injury

Required (single answer)

*Enabled conditional logic (rules to dynamically display or hide this field based on values from another field.): show this field if all of the following match:*

*Definition is “Injury”*

*Main cause of the injury is “Traumatic injury”*

**Contact injury**

1. Contact with another athlete
2. Contact: moving object (eg, boom)
3. Contact: immobile object (eg,cleats)

Required (multiple possible answers)

*Enabled conditional logic (rules to dynamically display or hide this field based on values from another field.): show this field if all of the following match:*

*Definition is “Injury”*

*Main cause of the injury is “Traumatic injury”*

*Traumatic injury is “Contact injury”*

**Contributing factors**

1. Recurrence of previous injury
2. Violation of rules (obstruction, pushing)
3. Field of play conditions
4. Weather condition
5. Equipment failure
6. Fatigue
7. Psychological
8. Other

Optional (multiple possible answers)

*Enabled conditional logic (rules to dynamically display or hide this field based on values from another field.): show this field if all of the following match:*

*Definition is “Injury”*

**Please, now consider your accident (series of events resulting in injury) and fill in the following fields.**

You should identify which factors can be changed and which changes would have the greatest impact on injury prevention. Fill in the boxes with all possible factors (risk and protective) that could have been involved in your accident.

|  | **Human factors** | **Boat and equipment factors** | **Environmental factors** |
| --- | --- | --- | --- |
| **Pre-Accident** |  |  |  |
| **Accident** |  |  |  |
| **Post Accident** |  |  |  |

**Do you have any further comments for the research team?**

|  |
| --- |

**I have read the the information sheet for the participant and I agree to take part to the present study.**

|  |
| --- |

Required

The electronic version is available at the following link: https://extremesportmed.org/survey/performance-test-for-the-foiling-week/
